# Anticandidal activities of lactic acid bacteria isolated from the vagina

**DOI:** 10.3906/sag-1709-143

**Published:** 2019-02-11

**Authors:** Sevda ER, Ayşe İSTANBULLU TOSUN, Gizem ARIK, Merih KIVANÇ

**Affiliations:** 1 Department of Medical Services and Techniques, Yunus Emre Vocational School of Health Services,Anadolu University, Eskişehir Turkey; 2 Department of Medical Microbiology, Faculty of Medicine, İstanbul Medipol University, İstanbul Turkey; 3 Department of Biology, Faculty of Sciences, Anadolu University, Eskişehir Turkey

**Keywords:** Agar diffusion technique, anticandidal activity, *Candida*, lactic acid bacteria

## Abstract

**Background/aim:**

Lactic acid bacteria prevent the overgrowth of pathogenic agents and opportunistic pathogens in the vagina. Moreover, lactic acid bacteria contribute to the preservation of vaginal microbiota by producing antimicrobial agents. Previous studies showed that some lactic acid bacteria exhibited antimicrobial activity against *Candida* species causing yeast vaginosis as well as many bacterial pathogens.

**Materials and methods:**

The antifungal activities of various lactic acid bacteria isolated from the vagina of healthy women on some *Candida* species isolated from the vagina were investigated by agar diffusion technique.

**Results:**

Most of the lactic acid bacteria that belong to the species of *Lactobacillus crispatus*,* L. fermentum*,* L. acidophilus*,* L. paracesei *subsp. *paracesei*, *L. pentosus*,* and L. plantarum* exhibited antifungal activity in varying ratios against *C. albicans*,* C. glabrata*, and* C. tropicalis* strains isolated from the vagina.

**Conclusion:**

The lactic acid bacteria are useful microorganisms associated with a variety of probiotic properties. In this sense, our lactic acid bacteria isolates with high antifungal activity may be promising candidates as probiotic microorganisms in the inhibition of vaginal candidiasis, which is one of the most prevalent problems, or in the protection against candidiasis. We will continue our studies in this area.

## 1. Introduction

Human microbiota colonized in human body consists of trillions of microorganisms. Different microbial communities were located in the vagina, mouth, skin, gastrointestinal tract, nose, urethra, and other parts of the body (1).

*Lactobacillus* species are dominant in the vaginal microbiota of healthy women (2). Premenopausal healthy women have 107–108 colony forming unit/gram lactobacilli in their vaginal fluid (3). Furthermore, *Staphylococcus* spp., *Ureaplasma* spp., *Corynebacterium* spp., *Streptococcus* spp., *Peptostreptococcus* spp., *Gardnerella vaginalis*, *Bacteroides* spp., *Mycoplasma* spp., *Enterococcus* spp., *Escherichia* *coli*, *Veillonella* spp., *Bifidobacterium* spp., and *Candida* spp. are also found in the vagina (4).

The lactobacilli in the vaginal microbiota protect the flora against the colonization of other sexually transmitted infectious agents such as bacterial vaginosis, urinary tract infections, vulvovaginal candidiasis, and AIDS. It was suggested that this is carried out through adhesion to the vaginal epithelium cells by competing with the pathogens and by producing antimicrobials such as bacteriocin, hydrogen peroxide, and lactic acid (5). 

Vulvovaginal candidiasis is a common infection seen in women throughout their lives. In some in vitro studies, some lactic acid bacterial strains were shown to inhibit the adhesion and development of *Candida albicans* (6). Drutz reported that oral administration of *Lactobacillus acidophilus* has a protective effect against *Candida* vaginitis (7). 

In the present study, it was aimed to investigate the anticandidal activities of lactic acid bacteria isolated from the vagina of the healthy women on the vaginal *Candida *isolates.

## 2. Material and methods

### 2.1. Isolation of microorganisms from the samples taken from the vagina

Lactic acid bacteria and yeast isolates were obtained through a physician from the samples taken from 30 healthy volunteer women aged between 20 and 40 years at the Gynecology Clinic of Medipol University Hospital, İstanbul. All were premenopausal and were not menstruating at the time of collection. All the studied women were clinically normal. They had not received antibiotics in the last 3 months. Vaginal samples were planted on the PDA (potato dextrose agar), chocolate agar, MRS (de Man, Rogosa, and Sharpe) agar, and M17 agar in the laboratory. For lactic acid bacteria isolation; MRS agar and M17 agar petri dishes were inoculated. The dishes were then incubated at 37 °C in 5% CO2 for 48–72 h. For yeast isolation, the PDA was inoculated and then the petri dishes were incubated at 30 °C for 5 days. Isolation of colonies developed in the petri dishes after incubation was performed. Primarily, Gram stains and catalase tests of the isolated microorganisms were performed. Gram-positive, catalase-negative bacterial isolates were separated as lactic acid bacteria and oxidase; mobility tests, development at different temperatures (4, 15, and 45 °C), development at different salt concentrations (6%, 7.5%, and 10% NaCl), development at different pH levels (pH 3.9 and 9.6), H2S formation, and ammonia formation from arginine were performed (8–9).

The microorganisms isolated from MRS agar, M17 agar, and PDA were stored at −85 °C. The permission of the ethical committee of our study was taken from the Ethics Committee of Non-Interventional Clinical Researches at the Istanbul Medipol University on Dec. 11, 04. All applicable international, national, and/or institutional guidelines for the care and use of human were followed.

#### 2.1.1. Determination of the use of carbohydrates with API CHL 50 by lactic acid bacteria isolates

The test was carried out by sowing API CHL 50 kits in accordance with the administrator’s instructions and the microorganisms were defined according to the carbohydrate sources they used. As a result of the test, the color change results of the isolates were entered into the database optimized by the management company and species identification was obtained as the % rates. 

#### 2.1.2. Definition of the yeast isolates by MALDI-TOF mass spectrometry

The colony sample was taken from the yeast isolate which was activated in PDA at 37 °C for 48 h and was planted on a 48-well plate. 0.3 μL of matrix solution was added onto the plate surface. In the device, the result obtained by performing the robust protein ionization and molecular weight measurement of the microorganism was based on the definition of microorganism by comparing it with the database. This part of the study was performed by BioMérieux Diagnostics Incorporated Company (Marcy-l’Étoile, France).

#### 2.1.3. 16srRNA sequence analysis

16s rRNA sequence analysis was performed for genotypic identification of the bacteria isolate. The genomic DNA of the isolate was purified using GeneJET genomic DNA purification kit (Thermo Fisher Scientific, Waltham, MA, USA). The obtained genomic DNA was used as template DNA and PCR reaction was performed for 16s rRNA gene locus. 27F 5’ AGAGTTTGATCMTGGCTCAG-3’ and 1492R 5’TACGGYTACCTTGTTACGACTT-3’ universal primers were used. PCR reaction components include 2.5 µL of 10X Taq buffer (+ KCl–MgCl2), 2.5 µL of 25 mM MgCl2, 2.5 µL of 2.5 mM dNTP mix, 2.5 µL of 2.5 mM 27F primer, 2.5 µL of 2.5 mM 1492R primer, 0.25 µL of Taq polymerase (5 U/µL), 11.75 µL of nuclease-free ddH2O, and 1 µL of template DNA. PCR products obtained from the reaction were screened in 1% agarose gel. 1492R and 907R (5’-CCGTCAATTCMTTTRAGTTT - 3’) primers were used for the sequence analysis of nearly 1400 base pair region (10). The sequence analysis of isolate was performed by MedSanTek Laboratory Supplies Trade & Industry Ltd (İstanbul, Turkey). 

#### 2.1.4. Determination of lactic acid production of the lactic acid bacteria isolates

The lactic acid bacteria isolate was incubated in the MRS broth medium at 37 °C and 5% CO2 for 48 h. Following the incubation, 1 mL of fresh lactic acid bacteria culture was transferred to a clean flask and filled up to 100 mL by sterile dH2O. Two or three drops of phenolphthalein indicator were added and titrated with 0.1 M NaOH solution. The amount of NaOH was recorded. Acid produced by the culture was calculated as percent titrable acidity. Lactic acid amount produced by the bacteria was calculated by the formula below. The study was conducted in duplicate (11).

Acidity %: 0.1 N NaOH (mL) amount used × 0.9/mL

#### 2.1.5. Determination of hydrogen peroxide production of the lactic acid bacteria isolates

5 mL of distilled water was added to the lactic acid bacteria cultures and centrifuged at 5000 rpm for 15 min. After centrifugation, the clear liquid formed on top was removed and filtered through Whatman No. 42 filter paper (Buckinghamshire, UK). After filtration, 4 mL of the obtained filtrate was taken into a separate tube. On top of this filtrate, 0.5 mL of sulfuric acid, 0.5 mL of ammonium molybdenum, and 0.5 mL of potassium iodide solution were added, and after each chemical addition, the samples were thoroughly vortexed. After all these processes were carried out, the optical densities of the obtained liquid were determined at 350 nm wavelength using a Shimadzu UV-1800 spectrophotometer (Kyoto, Japan). The obtained optical density (OD) values were calculated in terms of μg/mL according to the previously prepared standard curve (12).

### 2.2. Determination of anticandidal activity

Anticandidal activity was investigated by agar spot technique. For this purpose, the lactic acid bacteria were incubated in MRS and M17 broth for 48 h. After incubation, the lactic acid bacteria were adjusted to 0.5 McFarland turbidity in 0.85% physiological saline. 5 μL of the turbidity-adjusted lactic acid bacteria sample was instilled on the Mueller–Hinton soft agar containing glucose. After the petri dish was allowed to stand at room temperature for 30 min, it was allowed to incubate at 37 °C for 48 h.

After the incubation, the *Candida* isolates developed in potato dextrose broth at 37 °C for 48 h were adjusted to 0.5 McFarland turbidity in a 0.85% physiological saline solution. Potato dextrose soft agar inoculation was performed on the *Candida* isolates with turbidity adjustment. After the soft agar was thoroughly mixed, 7 mL of the mixture was slowly poured onto the surface of the petri dishes in which lactic acid bacteria were located. After the agar was frozen, the petri dishes were allowed to incubate at 37 °C for 48 h. After the incubation, zones around the lactic acid bacteria were observed and zone diameters were measured and recorded (13). The study was repeated twice.

## 3. Results

### 3.1. Isolation of microorganisms from the vaginal samples 

Lactic acid bacteria and yeast isolates were obtained from samples taken from 30 healthy females aged between 20 and 40 years at the Gynecology Clinic of Medipol University Hospital, İstanbul. It was made sure that the healthy women were not pregnant and that they had not used antibiotics in the last 3 months. It was demonstrated in the study that the 49 isolates isolated from MRS and M17 agars were catalase-negative (−), gram-positive (+). 14 isolates (8MR11, 13P1, 18P1, 19P3, 21P2, 24P1, 30P1, 14P1, 27P2, 13P2, 17P2, 16P1, 1C3, 5MR2 isolates), on the other hand, were found to be yeast. The isolates isolated from MRS, M17, potato dextrose, and chocolate agars were named to include “MR”, “M”,“P”, and “ C ” codes, respectively. 

In Table 1, the test results of 49 gram-positive (+) bacilli and catalase-negative (−) isolates for oxidase activity, growth at different temperatures, development at different salt concentrations, formation of hydrogen sulphide, and formation of ammonia from arginine are illustrated. According to these results, it was determined that all of the 49 isolates were oxidase-negative and immobilized. If we look at the development at different pH levels, 42 isolates developed at pH 3.9, while 7 isolates showed no improvement. Furthermore, 45 isolates showed a pH of 9.6 and 4 of them did not. Regarding the developments in different salt concentrations, 19 isolates were produced in the medium containing 6% NaCl and 30 isolates were not produced. 12 isolates were produced in the medium containing 7.5% NaCl, and 37 isolates were isolated. In the medium containing 10% NaCl, 9 isolates were produced and 40 isolates were not produced. If we look at the developments at different temperatures, 13 isolates grew at 4 and 15 °C and 36 did not show any growth. At 45° C, 43 isolates showed growth, 6 did not show any growth. As for the formation of hydrogen sulphide, no hydrogen sulphide or gas formation was observed in any of the 49 isolates. Ammonia was also formed from 18 isolate arginine and no ammonia was formed from 31 isolate arginine (Table 1).

**Table 1:** Biochemical activity tests of gram-positive (+) bacilli and catalase-negative (−) bacteria. (+): positive, (−): negative.

Microorganism	Isolate name	Oxidase	Mobility	Growing at pH 3.9	Growing at pH 9.6	Growing at 6% NaCl	Growing at 7.5% NaCl	Growing at 10% NaCl	Growing at 4 °C	Growing at 15 °C	Growing at 45 °C	H2S formatin	Ammonia formation from arginine
Lactobacillus crispatus	7MR5, 7MR7.4	(−)	(−)	(+)	(+)	(+)	(−)	(−)	(−)	(−)	(+)	(−)	(−)
	7MR1	(−)	(−)	(+)	(+)	(+)	(+)	(+)	(−)	(−)	(+)	(−)	(−)
	8MR19, 10MR3	(−)	(−)	(+)	(+)	(−)	(−)	(−)	(−)	(−)	(+)	(−)	(−)
	8MR20, 8MR4, 8MR9, 8MR3	(−)	(−)	(+)	(+)	(−)	(−)	(−)	(−)	(−)	(+)	(−)	(+)
	7MR4	(−)	(−)	(+)	(+)	(+)	(−)	(−)	(−)	(−)	(+)	(−)	(+)
	8MR1	(−)	(−)	(+)	(+)	(−)	(−)	(−)	(−)	(−)	(−)	(−)	(+)
Lactobacillus fermentum	5MR3, 5MR4	(−)	(−)	(+)	(+)	(+)	(+)	(+)	(+)	(+)	(+)	(−)	(+)
	1MR1.1, 5MR6	(−)	(−)	(+)	(+)	(+)	(+)	(−)	(+)	(+)	(+)	(−)	(−)
	11MR4	(−)	(−)	(+)	(+)	(−)	(−)	(−)	(−)	(−)	(+)	(−)	(−)
	5MR8, 5MR1	(−)	(−)	(+)	(+)	(−)	(−)	(−)	(−)	(−)	(+)	(−)	(+)
	11MR20	(−)	(−)	(+)	(−)	(−)	(−)	(−)	(−)	(−)	(+)	(−)	(−)
Lactobacillus acidophilus	10MR5, 10MR15, 10MR4,8MR2, 10MR14, 10MR6	(−)	(−)	(+)	(+)	(−)	(−)	(−)	(−)	(−)	(+)	(−)	(−)
	1MR3	(−)	(−)	(+)	(+)	(+)	(+)	(−)	(+)	(+)	(+)	(−)	(+)
Lactobacillus paracasei subsp. paracasei	10MR2, 8M1	(−)	(−)	(+)	(+)	(−)	(−)	(−)	(−)	(−)	(+)	(−)	(−)
	10MR7, 10MR8, 10MR9, 10MR18	(−)	(−)	(+)	(+)	(−)	(−)	(−)	(−)	(−)	(−)	(−)	(−)
	3M2, 3M6, 4M10, 4M5	(−)	(−)	(+)	(+)	(+)	(+)	(+)	(+)	(+)	(+)	(−)	(+)
	2M2	(−)	(−)	(+)	(+)	(+)	(+)	(+)	(+)	(+)	(+)	(−)	(−)
	10M7	(−)	(−)	(−)	(+)	(+)	(−)	(−)	(+)	(+)	(+)	(−)	(+)
	8M4	(−)	(−)	(+)	(−)	(−)	(−)	(−)	(−)	(−)	(+)	(−)	(−)
Lactobacillus pentosus	11MR12, 11MR19	(−)	(−)	(+)	(−)	(−)	(−)	(−)	(−)	(−)	(+)	(−)	(−)
	10M3, 10M10	(−)	(−)	(−)	(+)	(+)	(−)	(−)	(+)	(+)	(+)	(−)	(+)
	9M8	(−)	(−)	(−)	(+)	(−)	(−)	(−)	(−)	(−)	(+)	(−)	(−)
Lactobacillus plantarum	9M1, 9M3	(−)	(−)	(−)	(+)	(−)	(−)	(−)	(−)	(−)	(+)	(−)	(−)
	9M7	(−)	(−)	(−)	(+)	(+)	(−)	(−)	(−)	(−)	(+)	(−)	(−)
Lactobacillus delbrueckii subsp. delbrueckii	10MR12	(−)	(−)	(+)	(+)	(−)	(−)	(−)	(−)	(−)	(+)	(−)	(−)
	10MR13	(−)	(−)	(+)	(+)	(+)	(+)	(+)	(−)	(−)	(−)	(−)	(−)

MR: MRS agar, M: M17 agar. The bacteria isolated from MRS and M17 agars were named to include “MR” and “M” codes, respectively. NaCl: Sodium chloride, H2S: hydrogen sulphide.

#### 3.1.1. Determination of carbohydrate utilization status of the lactic acid bacteria isolates with API CHL 50 system

Identification results of 49 gram-positive (+) bacilli and catalase-negative (−) bacterial isolates isolated with the API CHL50 system are illustrated in Table 2. According to these results; of the 49 isolates, 8 were identified as *Lactobacillus acidophilus*, 3 as *Lactobacillus plantarum*, 9 as *Lactobacillus pentosus*, 8 as *Lactobacillus fermentum*, 17 as *Lactobacillus paracese*i subsp. *paracesei*, 13 as *Lactobacillus crispatus*, and 2 as *Lactobacillus delbrueckii* subsp. *delbrueckii*.

**Table 2:** Identification of lactic acid bacteria with API CHL 50 system.

Isolate name	API CHL 50 result	% of similarity
7MR4	Lactobacillus crispatus	99.9
7MR5, 7MR1,8MR9, 10MR3, 8MR3, 8MR4, 8MR1	L. crispatus	99.7
7MR7.4	L. crispatus	99.3
8MR19, 8MR20	L. crispatus	98.4
5MR3, 5MR1, 5MR8, 5MR4, 5MR6,11MR4, 11MR20	L. fermentum	95.3
1MR1.1	L. fermentum	88.9
10MR5, 10MR15, 10MR4, 10MR14	Lactobacillus acidophilus	97.4
1MR3	L. acidophilus	98.6
8MR2, 10MR6	L. acidophilus	95.3
10MR7, 4M5, 8M4	Lactobacillus paracesei subsp. Paracesei	99.9
10MR2, 10MR8, 3M2, 3M6, 10M7	L. paracesei subsp. paracesei	99.6
8M1	L. paracesei subsp. paracesei	97.7
10MR9, 10MR18	L. paracesei subsp. paracesei	80.5
2M2	L. paracesei subsp. paracesei	78.1
4M10, 11MR12, 11MR19	Lactobacillus pentosus	99.9
10M10, 10M3	L. pentosus	92.0
9M8	L. pentosus	88.9
9M3	Lactobacillus plantarum	99.2
9M7	L. plantarum	80.5
9M1	L. plantarum	72.6
10MR12, 10MR13	L. delbrueckii subsp. delbrueckii	92.8

MR: MRS agar, M: M17 agar. The bacteria isolated from MRS and M17 agars were named to include “MR” and “M” codes, respectively.

#### 3.1.2. Definition of yeast isolates by the MALDI-TOF mass spectrometry

By the MALDI-TOF Mass Spectrometry, 10 of 14 yeast isolates were *Candida albicans*, 3 was *C. glabrata*, and 1 was *C. tropicalis.*

#### 3.1.3. Results of the 16s rRNA sequence analysis 

The 5MR1, 5MR6, and 10MR5 isolates with high anticandidal activity were identified as *Lactobacillus jensenii* (GenBank accession no: MH327499*)*,* Enterococcus* *faecalis* (GenBank accession no: MH327502) with 99% similarity according to genotypic characterization results by 16S rRNA sequence analysis, and *L. jensenii* (GenBank accession no: MH327501), respectively.

#### 3.1.4. Lactic acid and hydrogen peroxide production of the lactic acid bacteria isolates

Table 3 illustrates the amounts of lactic acid and hydrogen peroxide in the lactic acid bacteria isolated from the vagina. While the lactic acid production of 49 isolates was between %0.91 and %2.684, it was found that the 10M7 isolate produced the lowest and the 5MR6 isolate produced the highest amount of lactic acid. As for the production of hydrogen peroxide, it was established that the production of hydrogen peroxide of 72 isolates was between 0.308 and 0.863 μg/mL. Furthermore, it was found that the 5MR8 isolate produced the lowest and 10M3 isolate produced the highest amount of hydrogen peroxide.

**Table 3:** The amounts of lactic acid and hydrogen peroxide of the lactic acid bacteria.

Microorganism	Isolatename	% of acidity	The amount of hydrogenperoxide (µg/mL)
L. crispatus	7MR5	2.366	0.629 ± 0.034
	7MR7.4	2.093	0.584 ± 0.166
	7MR1	2.184	0.680 ± 0.025
	8MR19	2.138	0.620 ± 0.064
	10MR3	2.184	0.473 ± 0.074
	8MR20	2.502	0.682 ± 0.019
	8MR4	1.82	0.502 ± 0.123
	8MR9	1.911	0.607 ± 0.123
	8MR3	1.41	0.406 ± 0.001
	7MR4	2.275	0.589 ± 0.000
	8MR1	1.547	0.705 ± 0.080
L. fermentum	5MR3	1.911	0.389 ± 0.106
	5MR4	1.956	0.572 ± 0.006
	1MR1.1	2.093	0.499 ± 0.199
	5MR6	2.684	0.555 ± 0.164
	11MR4	1.797	0.602 ± 0.079
	5MR8	2.002	0.308 ± 0.079
	5MR1	2.229	0.620 ± 0.060
	11MR20	2.093	0.633 ± 0.049
L. acidophilus	10MR5	2.366	0.663 ± 0.079
	10MR15	1.638	0.628 ± 0.077
	10MR4	1.911	0.773 ± 0.000
	8MR2	1.365	0.389 ± 0.030
	10MR14	1.82	0.597 ± 0.066
	10MR6	2.548	0.584 ± 0.043
	1MR3	1.41	0.569 ± 0.016
L. paracasei subsp.paracasei	10MR2	2.275	0.730 ± 0.000
	8M1	1.001	0.756 ± 0.019
	10MR7	1.274	0.392 ± 0.008
	10MR8	1.456	0.699 ± 0.068
	10MR9	1.683	0.607 ± 0.017
	10MR18	1.729	0.652 ± 0.071
	3M2	1.183	0.808 ± 0.040
	3M6	1.41	0.778 ± 0.022
	4M5	1.592	0.807 ± 0.025
	4M10	1.228	0.770 ± 0.026
	2M2	1.092	0.829 ± 0.030
	10M7	0.91	0.822 ± 0.059
	8M4	1.183	0.764 ± 0.036
L. pentosus	11MR12	1.865	0.672 ± 0.001
	11MR19	1.365	0.411 ± 0.000
	10M3	1.274	0.863 ± 0.096
	10M10	1.137	0.834 ± 0.027
	9M8	1.046	0.805 ± 0.046
L. plantarum	9M1	1.092	0.793 ± 0.001
	9M3	1.092	0.836 ± 0.034
	9M7	0.955	0.802 ± 0.047
L. delbrueckii subsp. delbrueckii	10MR12	1.638	0.707 ± 0.160
	10MR13	1.911	0.364 ± 0.041

MR: MRS agar, M: M17 agar. The bacteria isolated from MRS and M17 agars were named to include “MR” and “M” codes, respectively.

### 3.2. Anticandidal activities of the lactic acid bacteria isolates

Table 4 illustrates the anticandidal activity results of 49 lactic acid bacteria isolated and identified from the vagina against the 14 *Candida* species (*C. tropicalis *1C3,* C. glabrata *5MR2*, C. glabrata *16P,* C. glabrata *17P2,* C. albicans *8MR11*, C. albicans *13P1*, C. albicans* 13P2, *C. albicans* 14P1, *C. albicans *18P1,* C. albicans *19P3,* C. albicans *21P2*, C. albicans *24P1,* C. albicans *27P2,* C. albicans *30P) isolated from the vagina again. As far as the results are concerned, most of the lactic acid bacteria had variation of 8–44 mm of zone formation against the 14 *Candida* isolates. It was found that the lowest zone formation (8 mm) and the *L. acidophilus* 10MR14 isolate took place on the *C. albicans* 30P, and the highest zone formation (44 mm), on the other hand, *L. acidophilus* 10MR5 isolate took place on the *C. glabrata* 16P isolate.

**Table 4:** Anticandidal activities of the lactic acid bacteria.

Microorganism	Isolate name	1C3 (C. tropicalis)	5MR2 (C. glabrata)	16P (C. glabrata)	17P2 (C. glabrata)	8MR11 (C. albicans)	13P1 (C. albicans)	13P2 (C. albicans)	14P1 (C. albicans)	18P1 (C. albicans)	19P3 (C. albicans)	21P2 (C. albicans)	24P1 (C. albicans)	27P2 (C. albicans)	30P (C. albicans)
L. crispatus	7MR5, 7MR1,8MR4, 7MR7.4,8MR19, 8MR20	(−)	(−)	(−)	(−)	(−)	(−)	(−)	(−)	(−)	(−)	(+)	(+)	(−)	(−)
	10MR3	(+)	(−)	(−)	(−)	(−)	(−)	(−)	(−)	(−)	(+)	(+)	(+)	(+)	(+)
	7MR4	(−)	(−)	(−)	(−)	(−)	(−)	(−)	(−)	(−)	(−)	(++)	(−)	(−)	(−)
	8MR3, 8MR1, 8MR9	(−)	(−)	(++)	(−)	(−)	(−)	(−)	(−)	(−)	(−)	(+)	(+)	(+)	(−)
L. fermentum	5MR3	(+)	(−)	(−)	(−)	(−)	(−)	(−)	(+)	(+)	(+)	(+)	(+)	(+)	(+)
	1MR1.1, 11MR4	(−)	(−)	(−)	(−)	(−)	(−)	(−)	(−)	(−)	(−)	(+)	(+)	(+)	(−)
	5MR8, 5MR6, 5MR4, 5MR1	(−)	(−)	(++)	(−)	(−)	(−)	(−)	(−)	(−)	(−)	(++)	(+)	(+)	(−)
	11MR20	(+)	(−)	(−)	(−)	(+)	(+)	(−)	(+)	(+)	(+)	(+)	(+)	(−)	(+)
L. acidophilus	10MR5	(−)	(−)	(+++)	(−)	(−)	(−)	(−)	(−)	(−)	(−)	(+)	(++)	(+)	(−)
	10MR15, 10MR4	(−)	(−)	(++)	(−)	(−)	(−)	(−)	(−)	(−)	(−)	(+)	(+)	(+)	(−)
	8MR2	(−)	(−)	(++)	(−)	(−)	(−)	(−)	(−)	(−)	(−)	(+++)	(+)	(+)	(−)
	10MR14, 10MR6	(+)	(−)	(−)	(−)	(−)	(−)	(−)	(−)	(−)	(+)	(+)	(+)	(+)	(+)
	1MR3	(−)	(−)	(−)	(−)	(−)	(−)	(−)	(−)	(−)	(−)	(+)	(++)	(+)	(−)
L. paracasei subsp. paracasei	10MR2, 10MR7, 10MR8	(−)	(−)	(++)	(−)	(−)	(−)	(−)	(−)	(−)	(−)	(++)	(+)	(+)	(−)
	3M2, 3M6, 4M10	(+)	(−)	(−)	(−)	(−)	(+)	(+)	(−)	(+)	(+)	(+)	(+)	(−)	(+)
	2M2, 4M5, 8M1, 10M7	(+)	(−)	(−)	(−)	(+)	(+)	(+)	(−)	(+)	(−)	(+)	(+)	(+)	(−)
	8M4	(+)	(−)	(−)	(−)	(−)	(−)	(−)	(+)	(+)	(+)	(+)	(+)	(+)	(+)
	10MR9, 10MR18	(−)	(−)	(+)	(−)	(−)	(−)	(−)	(−)	(−)	(−)	(+)	(+)	(+)	(−)
L. pentosus	11MR12	(+)	(−)	(−)	(−)	(−)	(−)	(−)	(−)	(−)	(+)	(+)	(+)	(+)	(+)
	11MR19	(−)	(−)	(++)	(−)	(−)	(−)	(−)	(−)	(−)	(−)	(+)	(+)	(+)	(−)
	10M10, 10M3	(+)	(−)	(−)	(+)	(−)	(−)	(−)	(+)	(−)	(+)	(+)	(+)	(+)	(+)
	9M8	(+)	(−)	(−)	(−)	(+)	(+)	(−)	(+)	(+)	(+)	(+)	(+)	(+)	(+)
L. plantarum	9M3, 9M7	(+)	(−)	(−)	(−)	(+)	(+)	(−)	(+)	(+)	(+)	(+)	(+)	(+)	(+)
	9M1	(+)	(−)	(−)	(−)	(+)	(+)	(−)	(+)	(+)	(+)	(+)	(+)	(−)	(+)
L. delbrueckii subsp. delbrueckii	10MR12, 10MR13	(−)	(−)	(−)	(−)	(−)	(−)	(−)	(−)	(−)	(−)	(−)	(+)	(+)	(−)

The microorganisms isolated from MRS, M17, Potato dextrose, and chocolate agars were named to include “MR”, “M”,“P”, and “ C ” codes, respectively. Zone diameters are given in mm. (+):2 to ≥13 mm; (++) 14 to ≥25 mm; (+++) 26 to ≥38 mm.

## 4. Discussion

*Lactobacillus* species are dominant in the vaginal microbiota (14). It was reported that *Lactobacillus acidophilus*, *L. plantarum*, *L. casei*, *L. cellobiotus*, *L. oris, L. reuteri*,* L. ruminis*,* L. salivarius*,* L. brevis*,* L. delbrueckii*, and *L. vaginalis* species were commonly isolated from the vagina (15). The *Lactobacillus* in the vaginal microbiota protected the microbiota against the colonization of other sexually transmitted infections such as bacterial vaginosis, urinary tract infections, vulvovaginal candidiasis, and HIV (5). 

In the present study, anticandidal activities of various lactic acid bacteria isolated from the vagina of healthy volunteer women on the 14 vaginal *Candida* isolates were investigated. For this purpose, it was firstly determined that all the 49 lactic acid bacteria isolates were oxidase-negative and immobilized. It was observed that most isolates grew at pH 3.9 and pH 9.6. In addition, it was found that most isolates did not grow in medium containing 6%, 7.5%, and 10% NaCl and at 4, 15, and 45 °C. As for the formation of hydrogen sulphide, no hydrogen sulphide or gas formation was observed in any of the 49 isolates (Table 1). It was reported by Pektaş that not all of the 136 lactic acid bacteria isolates produced H2S (16). Similarly in our study, it was found that 18 of the 49 isolates did not form ammonia from arginine of 31 isolates, which formed ammonia from arginine (Table 1). It was reported that Lactobacilli such as *L. hilgardii*,* L. buchneri*, and *L. brevis* could hydrolyze the arginine (17). 

As a result of the identification of the isolated lactic acid bacteria by the API CHL 50 system, it was found that the 49 lactic acid bacteria isolates mostly belonged to *L. paracesei *subsp.* paracesei* (at the ratio of 27%) and to *L. crispatus* (at the ratio of 22.4%), followed by *L. fermentum* (16.3%), *L. acidophilus* (14.3%), *L. pentosus* (10.2%), *L. plantarum* (6.1%), and *L. delbrueckii* subsp. *delbrueckii* (4.1%) (Table 2). It was determined that 10 out of 14 yeast isolates isolated from the vagina by MALDI-TOF mass spectrometry belonged to *C. albicans *(8MR11, 13P1, 13P2, 14P1, 18P1, 19P3, 21P2, 24P1, 27P2, 30P), 3 isolates to *C. glabrata *(5MR2, 16P, 17P2), and 1 isolate to *C. tropicalis* (1C3). *Candida albicans* was the most common fungal species in the vaginal microbiota (18). In our study, *C. albicans* was also isolated at the highest level.

Vaginal homeostasis is protected by lactic acid and hydrogen peroxide production of the lactic acid bacteria in the vagina. By the production of lactic acid, the vaginal pH is kept below 4.5. It was reported that with the vaginal pH being acidic, it prevented the development of pathogens (19,20). In our study, the % acidity amounts of the lactic acid bacteria isolates isolated from the vagina ranged from 0.91 to 2.684 (Table 3). Furthermore, it was found that the hydrogen peroxide production amounts of our lactic acid bacteria isolates ranged from 0.308 ± 0.079 to 0.863 ± 0.096 μg/mL (Table 3). Halm et al. reported that the acidic medium formed by 2 *Lactobacillus reuteri* strains caused inhibition of yeasts (21). Alkali extracellular pH is thought to be the optimal pH for the development of *Candida* species; it was found that it induced the hyphae formation of *C. albicans*, and *C. albicans* remained in budding yeast form with less virulence at low pH (22). 

The *Candida *species found in the vaginal microbiota are opportunistic pathogens. These microorganisms in the vagina can cause infections in certain conditions (22). There are some studies in the literature on the anticandidal activities of the lactic acid bacteria. In these studies, it was reported that the lactic acid bacteria had anticandidal activity (23–26). In three of these studies (23–25), anticandidal activities of probiotics containing *Lactobacilllus *on *Candida albicans*,* C. glabrata*,* C. tropicalis, C. parapsilosis*, and *C. krusei* isolated from the vagina were investigated. In another study, the anticandidal activities of various vaginal *Lactobacillus* species on *Candida albicans* ATCC 32032, *Candida albicans* RTN 071, and *Candida glabrata* RTN 009 were studied (26). In our study, anticandidal activities of vaginal *Lactobacillus* spp. on vaginal *Candida* spp. were determined. Most of the vaginal lactic acid bacteria that belonged to the species of *L. crispatus*,* L. fermentum*,* L. acidophilus*,* L. paracesei *subsp*. paracesei*,* L. pentosus*, and* L. plantarum* exhibited anticandidal activity at varying ratios against vaginal C*. albicans*,* C. glabrata*, and* C. tropicalis* strains (Table 4). 5MR1, 5MR6, and 10MR5 isolates that had high anticandidal activity were identified by the 16S rRNA sequence analysis of *L. jensenii* (Accession no: AF243159.1), *Enterococcus faecalis* (Accession no. LC096215.1), and *L. jensenii* (Accession no: KF740715.1), respectively. It was found in this study that the lactic acid bacteria isolates had high amounts of lactic acid, and hydrogen peroxide had different antifungal activities. Besides, it was observed that the anticandidal activity was strain-specific. 

Lactic acid bacteria are useful microorganisms associated with a variety of probiotic properties (27). Recently, probiotics containing lactic acid bacteria with antibiotics have been used in the treatment of vaginal *Candida* infections (28). In conclusion, our *Candida* isolates with high anticandidal activity are promising so, further studies are needed to be able to use these strains as probiotics. Besides, no other study has been found in the relevant literature investigating anticandidal activity by isolating *Lactobacillus* and *Candida *from the vagina; therefore, this study will be of great contribution to the literature.

## Acknowledgments

This work was supported by a research grant from Anadolu University (research project 1305F089/2016). The authors would like to thank bioMérieux.
